# Genome-wide identification and expression analyses of phenylalanine ammonia-lyase gene family members from tomato (*Solanum lycopersicum*) reveal their role in root-knot nematode infection

**DOI:** 10.3389/fpls.2023.1204990

**Published:** 2023-06-06

**Authors:** Fulin Zhang, Juan Wang, Xianguo Li, Jun Zhang, Yuxiang Liu, Yijia Chen, Qinghui Yu, Ning Li

**Affiliations:** ^1^ Key Laboratory of Genome Research and Genetic Improvement of Xinjiang Characteristic Fruits and Vegetables, Institute of Horticultural Crops, Xinjiang Academy of Agricultural Sciences, Urumqi, China; ^2^ The State Key Laboratory of Genetic Improvement and Germplasm Innovation of Crop Resistance in Arid Desert Regions (Preparation), Institute of Horticultural Crops, Xinjiang Academy of Agricultural Sciences, Urumqi, China; ^3^ College of Horticulture, Xinjiang Agricultural University, Urumqi, China; ^4^ Comprehensive Proving Ground, Xinjiang Academy of Agricultural Sciences, Urumqi, China

**Keywords:** tomato, PAL gene family, root-knot nematode, bioinformatics, phylogenetic

## Abstract

Phenylalanine ammonia-lyase (PAL) is a key enzyme and rate-limiting enzyme of phenylpropanoid metabolism, which is a very important pathway in plants, and the secondary products it produces play an important role in plant growth and development, disease resistance, and stress resistance responses. However, PALs still lack systematic characterization in tomato. Based on a bioinformatics methods, PAL family genes were identified and characterized from tomato. qRT-PCR was used to study the expression of PAL genes in cultivated tomato after root-knot nematode infection. In this study, 14 and 11 *PAL* genes were identified in cultivated and wild tomatoes, and phylogenetic analysis classified them into three subfamilies, with different subfamilies of PAL proteins evolving in different directions in monocotyledonous and dicotyledonous plants. The extensive presence of stress, growth, hormone, and light response elements in the promoter sequences of *SlPAL* (*Solanum lycopersicum*) and *SpenPAL* (*Solanum pennellii*) genes suggests that this family has a critical role in abiotic stress. Collinearity indicates that members of the tomato and Arabidopsis *PAL* genes family are from the same ancestor, and the *SlPAL10* gene is directly homologous to monocotyledonous rice and maize, suggesting that the *SlPAL10* gene was present before monocotyledonous differentiation. Two co-expressed gene modules containing *PAL* genes were screened by WGCNA, and the core genes in the network were mined and functionally annotated by calculating the connectivity of genes within the modules. In addition, the expression of some genes changed significantly after root-knot nematode infection, with up-regulation of 4 genes and down-regulation of 3 genes. This result provides a data reference for the study of *PAL* family gene functions in tomato, and also provides a potential application for the subsequent selection of *PAL* genes in tomato for root-knot nematode resistance.

## 1 Introduction

Phenylalanine ammonia-lyase (PAL, EC 4.3.1.5), an intracellular inducible enzyme, is an acidic protein (Costa et al., 2003), which is a key and rate-limiting enzyme in the metabolism of phenylpropanoids and the most studied enzyme in the metabolic pathway of phenylpropanoids (Ritter and Schulz, 2004; MacDonald and D’Cunha, 2007). It is widely found in plants, fungi, yeast and algae, but has not been found in animals (Huang et al., 2010). Phenylpropanoid metabolism is a very important pathway in plant metabolism, and all substances containing the phenylpropanoid backbone are synthesized directly or indirectly by this pathway (Xu and Yang, 2009). Phenylpropanoid metabolism generates a variety of secondary metabolites such as flavonoids and lignans, which play important roles in plant growth and development, disease resistance, and resistance responses (Li et al., 2018; Deng et al., 2019; Dong and Lin, 2021). PAL genes play an important role in plant defense systems and are mainly involved in the biosynthesis of the signaling molecule salicylic acid, which is organic acid essential for the acquisition of resistance in plant systems (Nugroho et al., 2002; Chaman et al., 2003). PAL genes are found in a variety of plants, such as Arabidopsis (*Arabidopsis thaliana*) (Huang et al., 2010), aspen (Populus tremula) (Shi et al., 2013), banana (Musa nana) (Wang et al., 2016), rice (Oryza sativa) (Zeng et al., 2018) and walnut (Juglans rega) (Yan et al., 2019), among others.

Members of the plant PAL gene family respond differently to various hormones and stresses and are differentially expressed in different tissues and developmental stages of the plant body. The PAL gene family consists of four genes in Arabidopsis, AtPAL1-4, of which AtPAL1, AtPAL2, and AtPAL4 are highly expressed in the stem, and both AtPAL2 and AtPAL4 are expressed in the seed (Fraser and Chapple, 2011). In addition, the expression of AtPAL1 and AtPAL2 in leaves was induced by low temperature and nitrogen depletion (Olsen et al., 2008). There are 12 members of the PAL gene family in watermelon, of which 11 ClPAL genes are abundantly expressed in stems, male and female flowers, and 6 ClPAL genes are abundantly or moderately expressed in fruits, and their expression levels are regulated by ethylene signaling (Dong and Shang, 2013). Also PAL has good disease resistance, and overexpression of AevPAL1 in bread wheat (*Aegilops variabilis*) significantly enhanced resistance to the pathogen cereal cyst nematode (CCN) (Zhang et al., 2021). In wheat (*Triticum aestivum*), 11 PAL genes expression was up-regulated and 14 PAL genes expression was down-regulated after inoculation with Puccinia striiformis. The disease severity of TaPAL32 and TaPAL42 silenced plants was found to be higher than that of control plants 14 days after inoculation by VIGS (Zhan et al., 2022).

Tomato (*Solanum lycopersicum*) occupies an extremely important position in vegetable production in China and is characterized by high nutrient content and yield (Liu et al., 2022; Song et al., 2022). However, during the growth and development of tomato, it is highly susceptible to root-knot nematode (*Meloidogyne incognita*) infection, which seriously affects the yield and quality of tomato. The aim of this study was to identify the composition of PAL genes in cultivated tomato (*Solanum lycopersicum*) and wild tomato (*Solanum pennellii*) at the genome-wide level by bioinformatics methods and analyze their physicochemical properties, phylogenetic relationships, gene structure, covariance, promoter elements, and protein interactions. In order to analyze the expression regulation and function of tomato PAL gene family in root-knot nematode resistance, and to verify its expression pattern in root-knot nematode infection by qRT-PCR, and to provide clues for the discovery of excellent genetic resources and improvement of resistant varieties of tomato.

## 2 Materials and methods

### 2.1 Experimental materials

Southern root knot nematode (Meloidogyne incognita), collected from Langfang, Hebei, China, and single egg masses were picked for expansion and culture on susceptible tomatoes. Egg masses were manually picked from the root system and second stage juveniles at 25 °C were used for inoculation. Tomato ‘AC’ model variety, provided by the Tomato genetic breeding group, Institute of Horticultural Crops, Xinjiang Academy of Agricultural Sciences. Planted in the Key Laboratory of Genome Research and Genetic Improvement of Xinjiang Characteristic Fruits and Vegetables, the planting conditions were inoculated with 1000 second instar larvae of root-knot nematodes/plant in sterile sandy soil (7: 3) from tomato roots after 3-4 weeks. 5 days after inoculation, tomato roots were collected and four biological replicates were set up for each treatment and stored at -80°C after liquid nitrogen snap freezing for RNA Extraction.

### 2.2 Identification of tomato PAL protein sequences

Download the tomato 5.0 genome annotation file from the Solanaceae Genome Database (http://solomics.agis.org.cn/tomato/), and download the *S. pennellii* genome annotation file from the Solanaceae Database (https://solgenomics.net/). Four AtPAL amino acid sequences (Huang et al., 2010) were downloaded from the TIAR website (http://www.arabidopsis.org) based on known reports. AtPALs were used as genome-wide query sequences in the Phytozome (https://phytozome.jgi.doe.gov) database for BLASTP to obtain PAL members of rice, potato (*Solanum tuberosum*) and maize (*Zea mays*) (Goodstein et al., 2012; Fernandez-Pozo et al., 2015). Hidden Markov files (PF00221.21) of PAL structural domains were downloaded from the Pafm database (https://www.ebi.ac.uk/interpro/search/text/), and tomato and S pennellii candidate genes (E-value: 1e-5) were extracted using Hmmsearch (Eddy, 2008; Finn et al., 2011). The protein sequences of the Arabidopsis PAL family genes were used as seed sequences for local construction of BLAST libraries, which were analyzed using Smart (http://smart.embl.de/) and NCBI-CDD (https://www.ncbi.nlm.nih.gov/cdd) online software to validate the tomato and S. pennellii PAL family gene The tomato and *S. pennellii* PAL family genes were finally identified by deleting genes without typical PAL family domains (Sonnhammer et al., 1997; Schultz et al., 2000). The physicochemical properties of PAL genes were predicted by the online Expasy (https://web.expasy.org/protparam/) tool (Artimo et al., 2012) and by WoLF PSORT (https://www.genscript.com/wolf-psort.html?src=leftbar) online software to predict protein subcellular localization (Horton et al., 2007).

### 2.3 Chromosome localization, phylogenetic tree construction, gene structure and conserved motifs

Chromosome lengths and all tomato PAL family loci were obtained based on the genome annotation file GFF3 and then conformed using Map Inspect software (Jiang et al., 2019). The phylogenetic tree was constructed using MEGA7 software to analyze the PAL amino acid sequences of Arabidopsis, rice, maize, potato, tomato, and *S. pennellii* by multiple sequence alignment and set by Neighbor-joining (NJ) and Maximum likelihood (ML), Bootstrap test 2000 replicates (Kumar et al., 2016). Itools online website (https://itol.embl.de/) was used to display the midpoint root tree. Mapping of intron exons was performed in TBtools software using existing tomato gff files and tomato PAL gene family IDs to map the tomato PAL gene structure. motif was then obtained using meme software to obtain the meme.xml file of the *SlPAL family genes*, with the search motif value set to 20 and other default parameters (Grundy et al., 1997). The conserved motifs were then predicted for analysis using Visualize MEME/MAST Motif Pattern in TBtools software (Chen et al., 2020). Structural domain analysis was performed using the SlPALs protein sequence in NCBI-cdd to obtain hit data files, which were plotted by TBtools software.

### 2.4 Cis-regulatory elements, collinearity and interaction network analysis

The 2000 bp sequence upstream of all tomato PAL genes was extracted as candidate promoter sequences using tomato genome annotation files, cis-acting element analysis was performed using PlantCare (http://bioinformatics.psb.ugent.be/webtools/plantcare/html/) website (Rombauts et al., 1999), and cis-acting element mapping was performed using TBtools software (Chen et al., 2020). For multispecies covariance, MCscanX in TBtools software was used to extract covariate genes of species pairs and finally mapped using Multiple Synteny Plot. The substitution rate of paralogous genes was calculated using KaKs_Calculator 2.0 (Wang et al., 2010). Protein-protein interaction relationships were predicted using the STRING online website (https://string-db.org/) (Szklarczyk et al., 2019).

### 2.5 Validation of tomato *PAL* family genes by qRT-PCR

Plant root RNA was extracted using the Tiangen Plant Polyphenol Polysaccharide Total RNA Extraction Kit (Beijing, China). The extracted total RNA was then subjected to cDNA synthesis using the 5 × All-ln-One RT MasterMix (AccuRT Genomic DNA Removal Kit; G492, ABM, Vancouver, Canada) reverse tanscription kit. qRT-PCR primers for *SlPAL* family members were designed using DNAMAN6 software. The designed qRT-PCR primers were sent to Biotech Biologicals (Shanghai, China) for synthesis (**Supplementary Table S1**). Quantitative PCR (qPCR) analysis was subsequently performed on a LightCycler machine using ChamQ Universal SYBR qPCR Master Mix (Q711, Vazyme, Nanjing, China). The Slactin was used as the internal reference gene. Each treatment contains four independent biological replicates, and each replicate contains three technical replicates. Gene expression was calculated using the 2^-ΔΔCt^ method (Livak and Schmittgen, 2001).

## 3 Results

### 3.1 Identification and physicochemical property analysis of tomato PAL family members

In this study, we used HMM search and Arabidopsis amino acid sequences (AtPALs) for BLAST. The results were finally combined, redundant sequences were removed, and they were named according to their positions on the chromosomes (**Table 1**). Finally, 14 members of the cultivated tomato PAL family gene were identified and named SlPAL1-SlPAL14, and the 11 members of the wild relatives of S. pennellii PAL family gene were named SpenPAL1-SpenPAL11. Two tomato PAL family amino acids range in length from 218 (SpenPAL4) to 1274 (SlPAL10). Molecular weights were in the range of 23065.33 (SpenPAL4)-139461.81 (SlPAL10). pI ranged from 5.61 (SlPAL3)-9.44 (SpenPAL4). The pI values of 11 PAL genes of cultivated tomatoes were less than 7, and the pI values of 10 genes PAL genes of S. pennellii were also less than 7, indicating that most of these two tomatoes belong to acidic proteins. The pI values ranged from 78.17 (SlPAL6) to 104.10 (SlPAL5), hydrophobic values ranged from 0.055 to -0.432, only SlPAL5 was 0.055, and the rest of the PAL family hydrophilic values of cultivated tomatoes and S. pennellii were less than zero, indicating that these proteins are hydrophobic. In subcellular localization, it can be found that there are 8 SlPAL genes distributed in chloroplasts, one in cytoplasm of 3 distributions, one in mitochondria, plasma membrane and endoplasmic reticulum respectively, 6 SpenPAL genes distributed in chloroplasts, two in cytoplasm and endoplasmic reticulum respectively, and one in plasma membrane.

**Table 1:** Identification of *SlPAL* and *SpenPAL* family gene members and analysis of their physicochemical properties.

Gene id	Gene name	Chr	Length	MW(Da)	PI	Aliphatic index	GRAVY	Subcellular localization
*Solyc03T000684.1*	*SlPAL1*	3	630	68901.67	5.99	89.95	-0.224	Cyto
*Solyc03T000693.2*	*SlPAL2*	3	692	75395.2	6.00	94.29	-0.160	Chlo
*Solyc03T000697.1*	*SlPAL3*	3	543	59886.22	5.61	88.73	-0.143	Plas
*Solyc03T000702.1*	*SlPAL4*	3	692	75444.46	6.09	94.42	-0.136	chlo
*Solyc03T001018.1*	*SlPAL5*	3	547	60017.4	7.03	104.10	0.055	chlo
*Solyc03T001540.1*	*SlPAL6*	3	120	13237.17	7.13	78.17	-0.432	Mito
*Solyc03T001541.1*	*SlPAL7*	3	258	28781.8	5.91	98.18	-0.238	cyto
*Solyc05T002733.1*	*SlPAL8*	5	709	77461.52	6.19	92.89	-0.193	E.R.
*Solyc09T000189.7*	*SlPAL9*	9	828	89507.43	6.81	90.36	-0.158	chlo
*Solyc09T000190.2*	*SlPAL10*	9	1274	139461.81	7.22	91.41	-0.133	cyto
*Solyc10T000519.1*	*SlPAL11*	10	709	77497.62	6.00	93.67	-0.152	chlo
*Solyc10T000520.1*	*SlPAL12*	10	709	77581.59	5.85	93.54	-0.157	chlo
*Solyc10T000521.1*	*SlPAL13*	10	709	77581.59	5.85	93.54	-0.157	chlo
*Solyc10T002904.3*	*SlPAL14*	10	1016	111251.95	5.72	88.52	-0.170	chlo
*Sopen03g007350.1*	*SpePAL1*	3	715	77834.16	6.08	94.28	-0.129	E.R.
*Sopen03g007390.1*	*SpenPAL2*	3	421	45509.19	6.84	90.40	-0.135	plas
*Sopen03g021000.1*	*SpenPAL3*	3	313	34816.71	6.12	95.91	-0.267	cyto
*Sopen03g021010.1*	*SpenPAL4*	3	218	23065.33	9.44	88.58	-0.059	cyto
*Sopen05g034580.1*	*SpenPAL5*	5	710	77547.48	6.03	92.48	-0.200	E.R.
*Sopen09g002720.1*	*SpenPAL6*	9	722	78668.96	6.29	92.94	-0.186	chlo
*Sopen09g002730.1*	*SpenPAL7*	9	703	76581.35	6.29	90.46	-0.215	chlo
*Sopen09g002740.1*	*SpenPAL8*	9	724	78696.93	6.04	91.20	-0.171	chlo
*Sopen09g002750.1*	*SpenPAL9*	9	724	78739.09	6.39	92.00	-0.166	chlo
*Sopen10g005710.1*	*SpenPAL10*	10	709	77600.58	6.00	93.27	-0.173	chlo
*Sopen10g035560.1*	*SpenPAL11*	10	711	77484.4	5.83	92.62	-0.153	chlo

### 3.2 Chromosomal localization gene structure of tomato PAL family members

**Figure 1:**
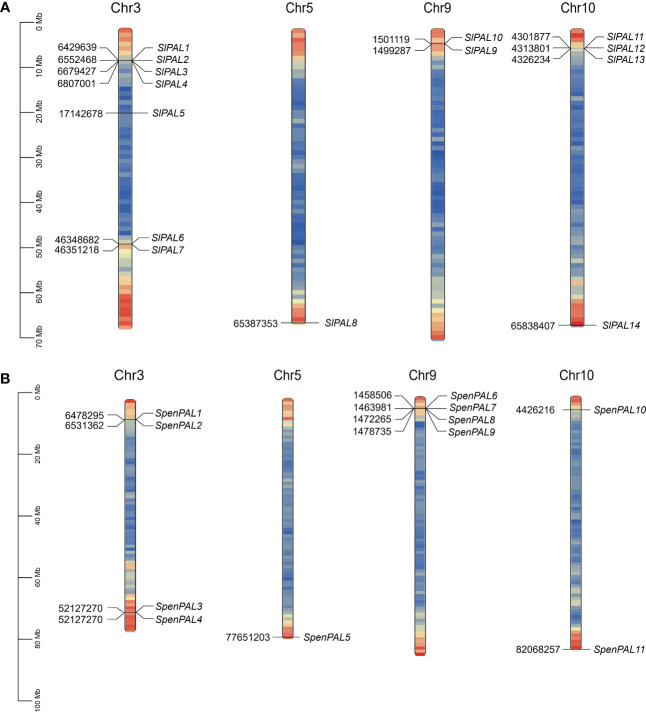
Chromosomal localization map of *SlPAL* and *SpenPAL* family gene members **(A)**: Chromosomal localization of *Solanum lycopersicum PAL* genes; **(B)**: Chromosomal localization of *Solanum pennellii PAL* genes.

To fully understand the gene distribution mechanism of the tomato PAL family on chromosomes (**Figure 1**), 14 SlPAL genes and 11 SpenPAL genes were mapped by TBtools, all distributed on chromosomes 3, 5, 9, and 10, respectively. And the *SlPAL* and *SpenPAL* families have 4 and 3 gene clusters, respectively, indicating that tandem replication is likely to be the main amplification mechanism of these two tomato gene families.

### 3.3 Multi-species PAL family phylogenetic tree construction

**Figure 2:**
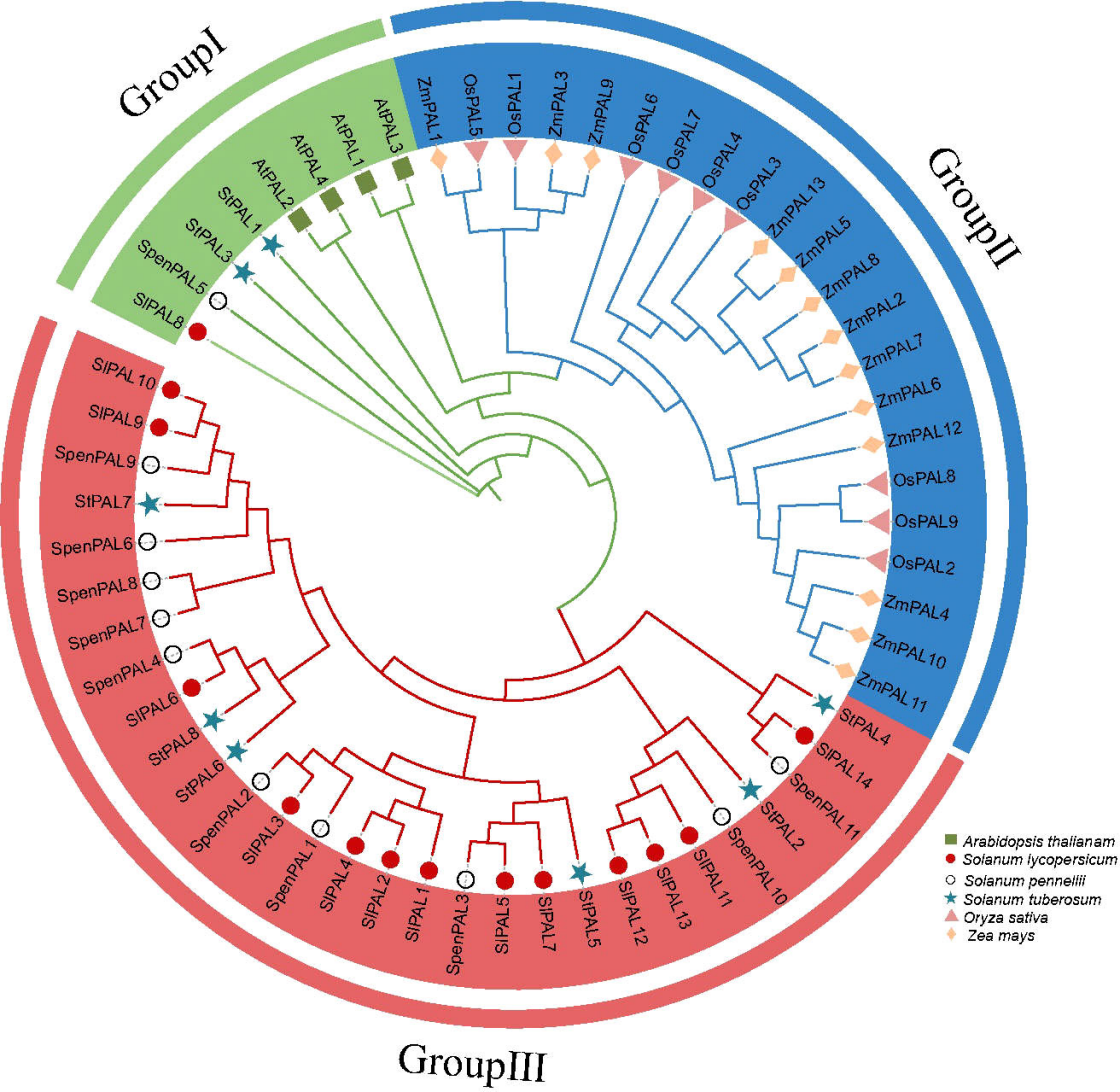
Phylogenetic analysis of multispecies PAL family proteins Phylogenetic trees were constructed for 59 *PAL* genes using the NJ method with 2000 bootstrap replicates. Different shapes represent different species. Different colors in the circle diagram represent different subclades.

To investigate the evolutionary relationships of PAL genes, phylogenetic analysis was performed on Arabidopsis (4), rice (9), maize (13), potato (8), cultivated tomato (14), and *S. pennellii* (11) altogether PAL genes (**Figure 2**). A phylogenetic tree diagram was constructed by comparison using MEGA 7.0 software. These PAL genes were clustered into three subclades (GroupI, GroupII, GroupIII). From the figure, we can find that monocotyledons and dicotyledons are more distantly related, and monocotyledons are all clustered on Group II subfamily, and PAL proteins of these subfamilies may evolve in different directions in monocotyledons and dicotyledons. The dicotyledonous plants potato, cultivated tomato, and S. pennellii occupy two subfamilies, Group I and Group III, thus confirming that the PAL genes of these three species are very inwardly related and may have similar biological functions.

### 3.4 Analysis of tomato *PAL* family gene structure, conserved motifs and structural domains

**Figure 3:**
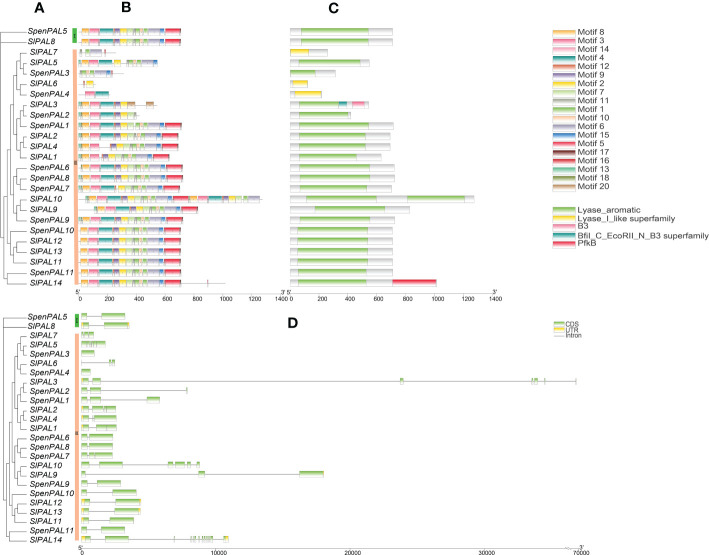
Conserved motifs, structural domains, gene structures of *SlPAL* and *SpenPAL* family gene **(A)**: Phylogenetic trees were constructed for the *PAL* genes of *Solanum lycopersicum* and *Solanum pennellii* using the NJ method with 2000 bootstrap replicates; **(B)**: Conserved motifs of *SlPAL* and *SpenPAL* genes, the numbers 1-20 and the different colored boxes indicate motifs; **(C)**: Structural domains of *SlPAL* and *SpenPAL* genes, different colors indicate different structural domains; **(D)**: Gene structures of *SlPAL* and *SpenPAL* genes, green boxes indicate exons, yellow boxes indicate UTRs, and black lines indicate introns.

To further understand the differences within the tomato PAL family, mapping was performed by TBtools software (**Figure 3**). The results showed that there were five structural domains in tomato PAL family, Lyase_aromatic, Lyase_I_like superfamily were the typical structural domains. It can be found that the tomato PAL family with Lyase_aromatic structural domains are also relatively uniform in their conserved motifs. Group III subfamily SlPAL10 has two Lyase_aromatic and its conserved motifs are duplicated, SlPAL6, SlPAL7, SpenPAL4 have the structural domains of Lyase_I_like the conserved motifs and structural domains of SlPAL5 and SlPAL8 of Group I subfamily are identical.

The intron (Intron), coding region (CDS), and untranslated region (UTR) of 14 SlPAL and 11 SpenPAL gene families were mapped using TBtools. 11 SpenPAL genes had 1-3 CDSs, 6 of which had 2 CDSs, but none of the 11 SpenPAL genes had a UTR. SlPALs genes have 2-13 CDSs, and SlPAL14 gene has the most CDSs. Ten of the SlPALs genes had UTRs, and five had two UTRs.

### 3.5 Comparison of tomato PAL family sequences using multiple sequence alignment

**Figure 4:**
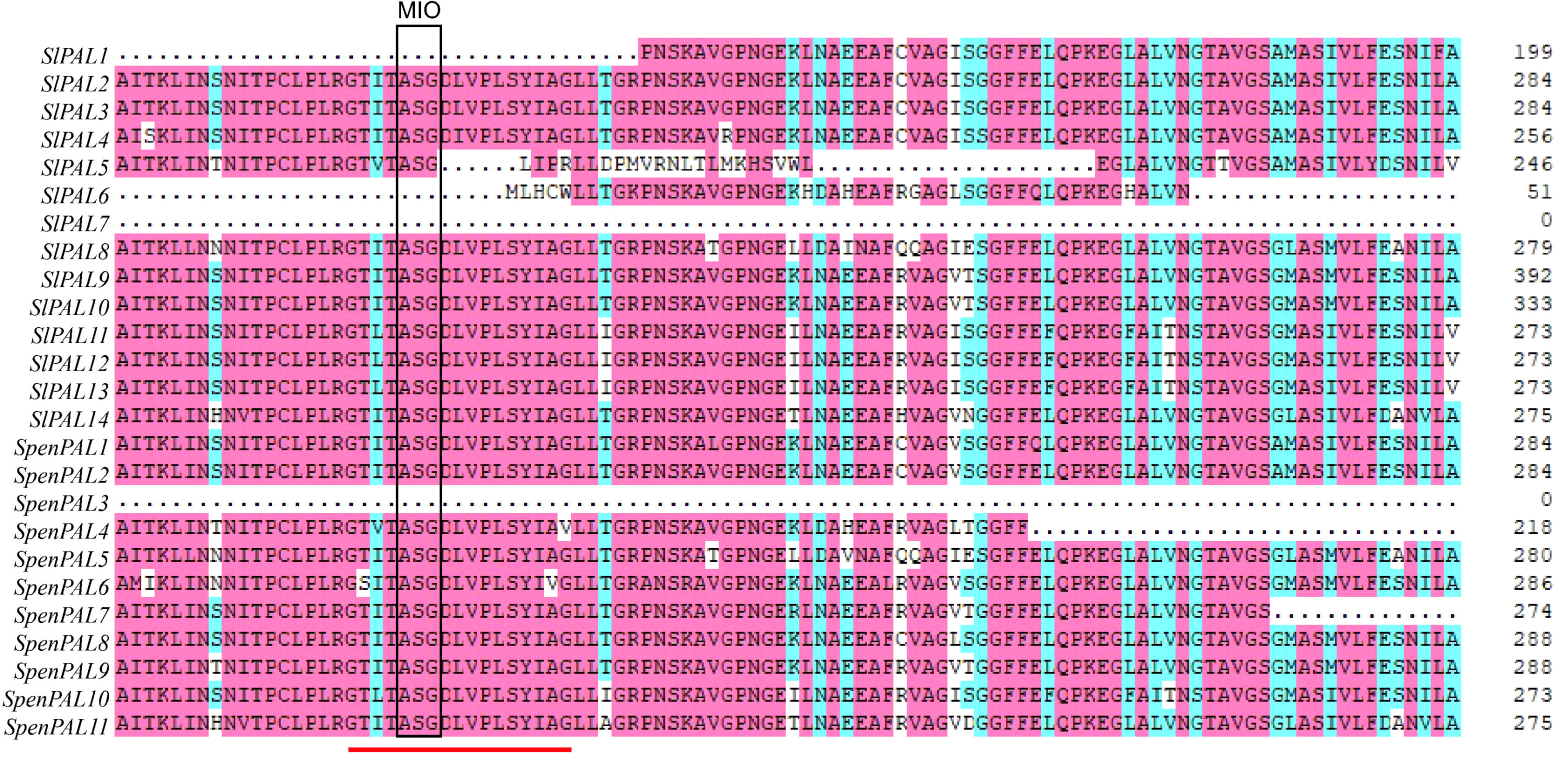
Multiple amino acid sequence alignment of *SlPAL* and *SpenPAL* gene families The red line indicates the active site of PAL. The box shows the electrophilic groups composed of Ala Ser Gly amino acid residues in PAL protein.

Multiple sequence alignment analysis revealed (**Figure 4**) that the amino acid sequences of both SlPAL and SpenPAL genes contain the active site GTITASGDLV(L)PLSYIAG of PAL, except for SlPAL1, SlPAL5, SlPAL6, SlPAL7, and SpenPAL3. In this site is contains the Ala-Ser-Gly composition of the highly conserved methylene imidazolone (MIO) electrophilic group. In the active sites of SlPAL5 and SpenPAL4, isoleucine (I) at position 3 may be mutated to valine (V), and glycine (G) at position 17 may be mutated to arginine (R) and valine (V). In the active sites of SlPAL11, SlPAL12, SlPAL13, and SpenPAL10, isoleucine (I) at position 3 may be mutated to leucine (L). The alanine (A) at position 16 in the active site of SpenPAL6 may be mutated to valine (V).

### 3.6 Promoter analysis of PAL genes in tomato

**Figure 5:**
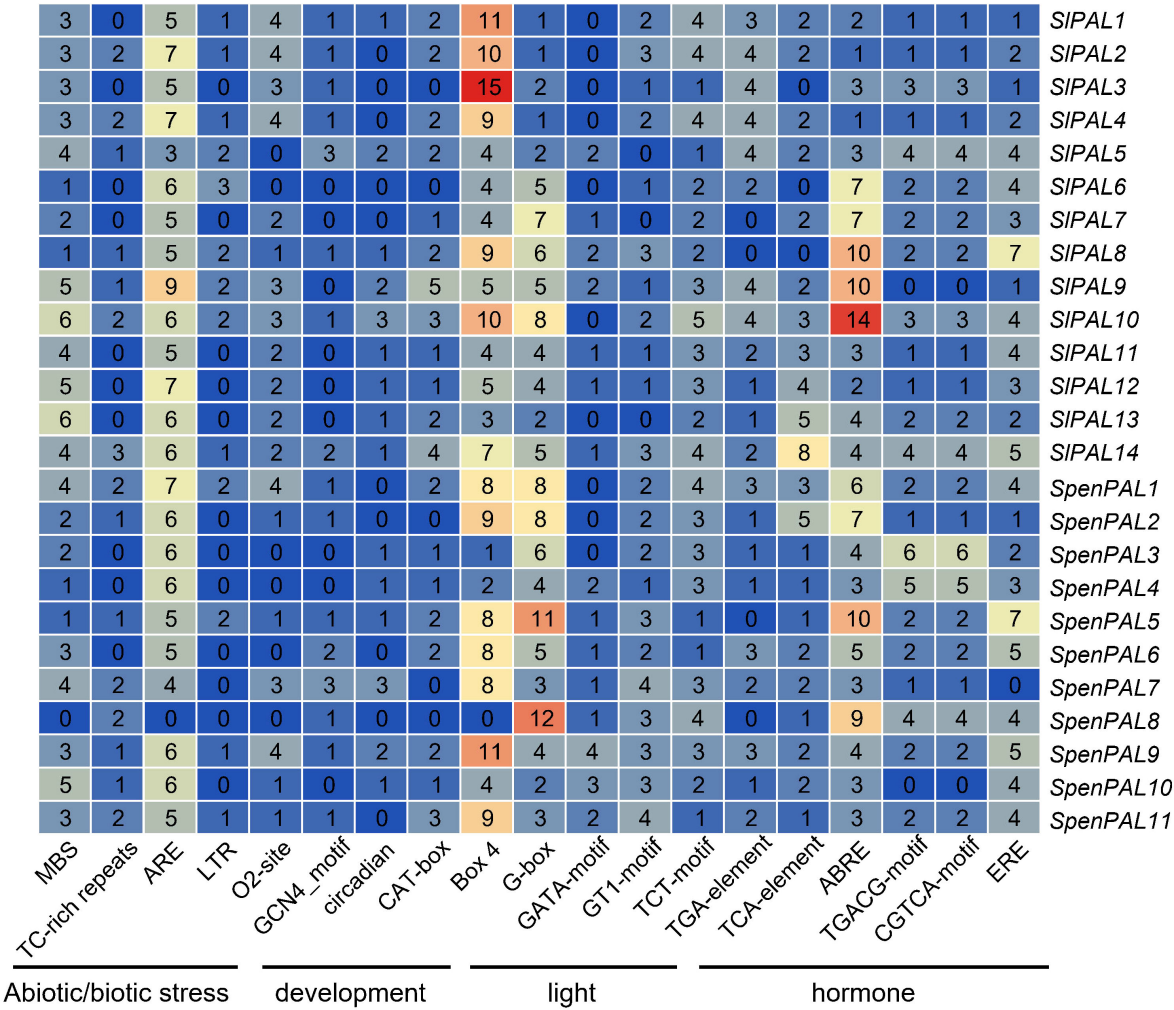
*Cis*-acting elements analysis of the promotors of *SlPALs* and *SpenPALs* genes Number: total number of elements found from the 2000 bp promoter region of the start codon; Color: from blue to red indicates the number of elements from small to large. The distance of the black line indicates elements of the same type.

Many studies have shown that the PAL gene family is involved in various stress responses. To better understand and elucidate the possible regulatory functions of SlPALs and SpenPALs under different stresses, we identified stress-related and phytohormone-related cis-elements from the 2000 bp promoter region of the start codon (**Figure 5**).

According to the data obtained from PlantCARE, five stress response control elements were found in the promoters of SlPALs and SpenPALs: anaerobic induction (ARE), drought (MBS), defense and stress response (TC-rich repeats), cold stress (LTR), and hypoxia-specific enhancers (GC-motif). 6 major growth and developmental response control elements were identified: regulation of maize protein metabolism (O2-site), involvement in endosperm expression (GCN 4 _ motif), participation in circadian control (circadian), participation in meristem expression (CAT-box), participation in cell cycle regulation (MSA-like), and participation in palisade mesophyll cell differentiation (HD-Zip 1). All found 20 major photoresponse elements: (AT1-motif, Sp 1, LAMP-element, GT1-motif, G-Box, ATC-motif, ATCT-motif, Box 4, MRE, AE-box, TCC-motif, G-box, TCT-motif, chs-CMA 1 a, ACE, CAG-motif, 3-AF1 binding site, AAAC-motif, GATA-motif, chs-CMA1a). 4 hormone-responsive elements were found: phytoauxin (TGA-element), gibberellin (P-box, TATC-box, GARE-motif), methyljasmonic acid (TGACG-motif, TCA-element, CGTCA-motif), and abscisic acid (ABRE). The heat map made to select the largest number of control elements in stress, growth, light response, and hormones shows that ARE elements in stress, O2-site elements in growth and development, Box 4 and G-box elements in light response, and ABRE elements in hormones are generally more abundant in SlPALs and SpenPALs, while SlPALs and SpenPALs also contain a large number of other regulatory elements. These results suggest that PAL genes are widely involved in a variety of life activities such as plant growth and development and stress response.

### 3.7 Multi-species *PAL* gene collinearity analysis

**Figure 6:**
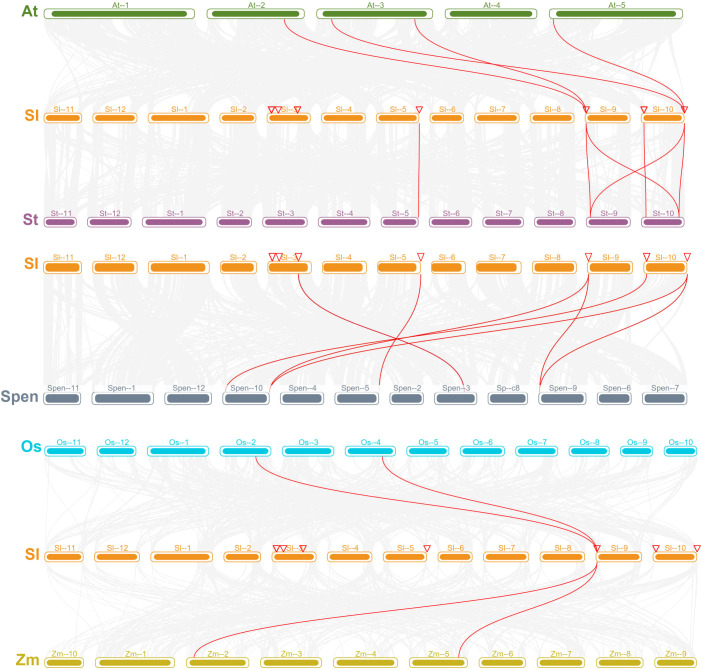
Interspecific collinearity analysis of *SlPAL* and *SpenPAL* family genes At, *Arabidopsis thaliana*; Sl, *Solanum lycopersicum*; St, *Solanum tuberosum*; Spen, *Solanum pennellii*; Os, *Oryza sativa*; Zm, *Zea mays*. From top to bottom, the *PAL* genes of dicotyledons directly homologous to tomato, the *PAL* genes of *Solanum pennellii* directly homologous to tomato, and the *PAL* genes of monocotyledons directly homologous to tomato.

To reveal the evolutionary origin and direct homology of the tomato PAL gene family, a covariance analysis of the PAL gene family on the chromosomes of four dicots [tomato (*S. lycopersicum*, *S. pennellii*), Arabidopsis, and potato] and two monocots (maize and rice) was performed in this study (**Figure 6**). The results showed that two genes of SlPALs were direct homologs with AtPALs, among which SlPAL10 had colinearity with AtPAL1 and AtPAL3, and SlPAL14 had colinearity with AtPAL2 and AtPAL4. There are 6 genes of SlPALs that are orthologous with SpenPALs, among which, SlPAL10 has collinearity with SpenPAL6 and SpenPAL11, SlPAL14 has collinearity with SpenPAL6 and SpenPAL11, and the collinearity of other genes corresponds. SlPALs have 1 gene that is direct homolog to OsPALs, but SlPAL10 has colinearity with OsPAL1 and OsPAL5. SlPALs also have 1 gene that is direct homolog to ZmPALs are direct homologs, but also SlPAL10 has colinearity with ZmPAL3 and ZmPAL9. For example, two genes, AtPAL1 and AtPAL3, have colinearity with SlPAL10, suggesting that the two pairs of genes may regulate similar or complementary functions, respectively. It can be found that most PAL genes in Arabidopsis and S. pennellii potato, which are dicotyledons, are direct homologs of the two genes SlPAL10 and SlPAL14. Interestingly, the PAL genes of rice and maize, two monocotyledons, are only direct homologs of SlPAL10, suggesting that the SlPAL10 gene existed before monocotyledon differentiation.

### 3.8 Duplication gene and Ka/Ks analysis of *PAL* genes in tomato

**Figure 7:**
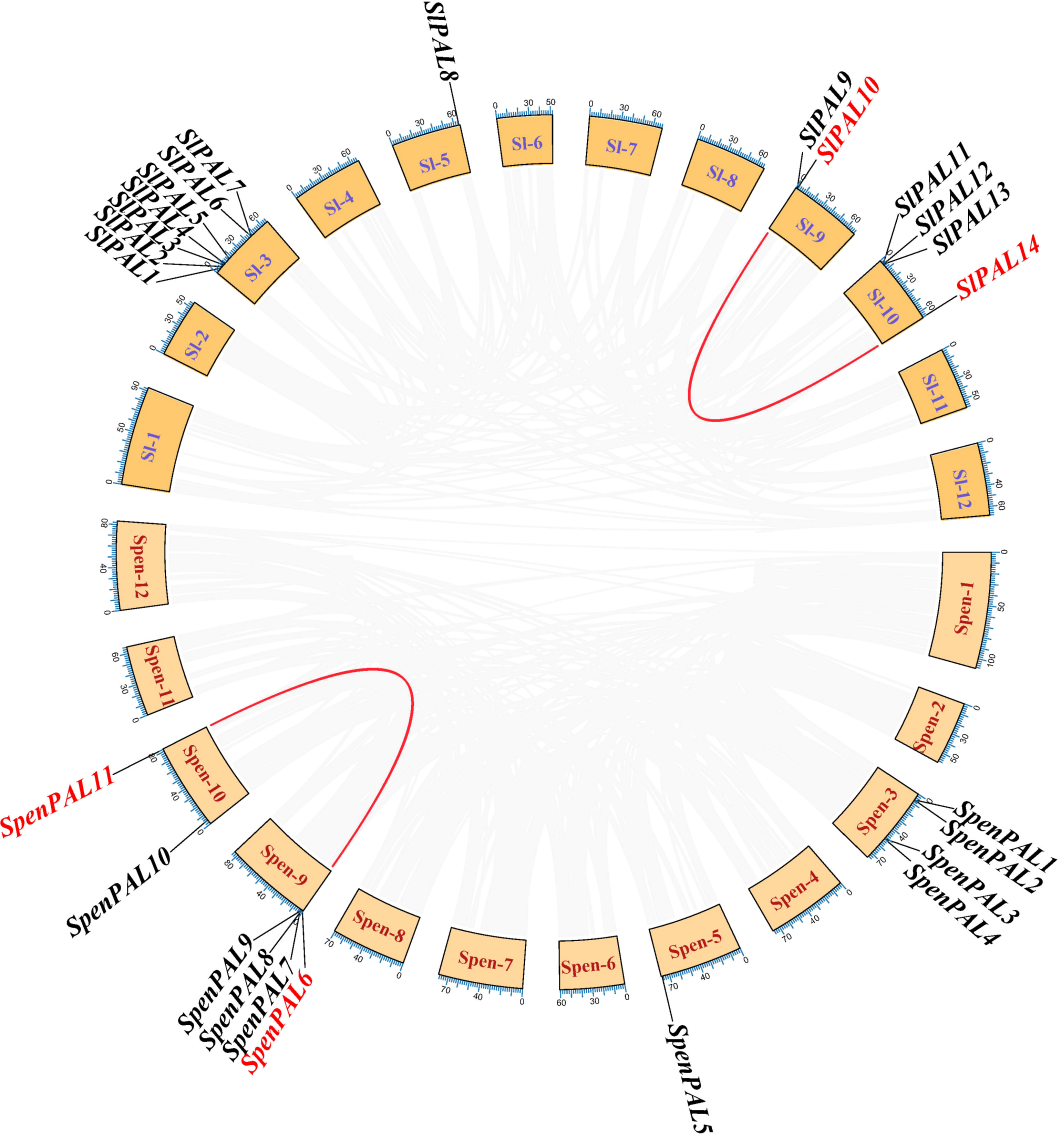
Intraspecific collinearity analysis of *SlPAL* and *SpenPAL* family genes Sl, *Solanum lycopersicum*; Spen, *Solanum pennellii*. Two species were analyzed for intraspecific homologous *PAL* genes. Red lines indicate homologous genes.

Repeated sequences on the tomato chromosome set were extracted by native BLAST and McscanX software to explore the co-linearity of the PAL gene family within the S. pennellii genome (**Figure 7**). As shown in the figure, there is one paralogous homologous gene pair each in cultivated tomato and S. pennellii, SlPAL10/SlPAL14 and SpenPAL6/SpenPAL11, and both of these paralogous homologous gene pairs originate from duplication of segments on their respective chromosomes 9 and 10.

The Ka/Ks ratio is usually an important indicator of selection pressure in evolution (**Table 2**). The ka/ks values of SlPAL10/SlPAL14 and SpenPAL6/SpenPAL11 paralogous homologous gene pairs were calculated by TBtools to be less than 0.3, indicating that the evolution of these two gene pairs was affected by purifying selection pressure, and the gene functions tended to be conserved and the evolutionary rate was slowed down.

**Table 2:** Repeat *SlPALs* and *SpenPALs* gene ka/ks values.

Species	Duplicated gene pairs	Ka	Ks	Ka_Ks	Selective pressure	Type
*S. lycopersicum*	*Solyc10T002904.3*/*Solyc09T000190.2*	0.205938	1.137032	0.181119	Purify selection	Segmental
*S. pennellii*	*Sopen09g002720.1*/*Sopen10g035560.1*	0.072051	0.831968	0.086603	Purify selection	Segmental

### 3.9 Protein–protein network analysis of *PAL* family genes in tomato

**Figure 8:**
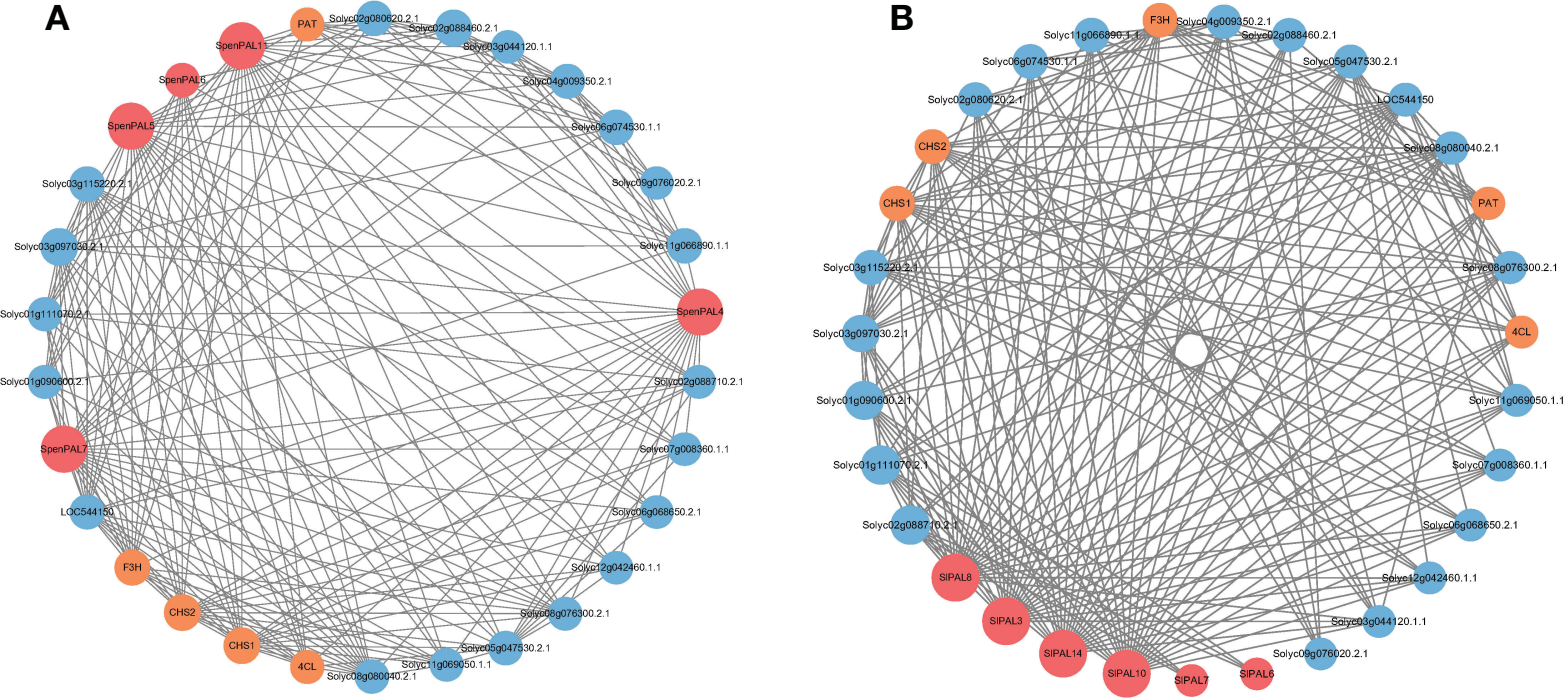
*SlPAL* and *SpenPAL* family genes protein interactions **(A)**: *Solanum lycopersicum*; **(B)**: *Solanum pennellii*. Each node is a protein and the presence of each edge representing an interaction node indicates the number of interactions, i.e. the thickness of the edge indicates the value of the combined score, with red nodes representing PAL proteins, yellow nodes representing stress-related proteins, and blue nodes representing proteins lacking annotation.

The mechanism of action of different tomato PAL proteins was specifically analyzed by constructing the interaction network of SlPALs and SpenPALs proteins (**Figure 8**). We excluded some proteins with missing annotations and low values, and the results showed that the highest number of proteins interacted with SlPAL8, SlPAL3, SlPAL14 and SlPAL10 in cultivated tomato and they had strong interactions with each other. Among them, SlPAL6 and SlPAL7 had no direct interactions with other PAL proteins. In contrast, the highest number of proteins interacted with SpenPAL4, SpenPAL5, SpenPAL7 and SpenPAL11 in S. pennellii. Among them, there were no direct interactions between SpenPAL6 and other PAL proteins. As can be seen from the figure, different tomatoes exhibited similar protein action mechanisms. Chalcone synthase (CHS) is the first rate-limiting enzyme in the plant flavonoid biosynthetic pathway, and the expression of CHS is closely related to the anthocyanin synthesis and accumulation metabolism. The CHS gene is also involved in plant response to abiotic stresses, with implications for plant flower color formation, growth and development, adversity stress, hormone regulation and transport (Dao et al., 2011). Flavonoid 3’-hydroxylase (F3’H), an intermediate in the synthesis of anthocyanins, often cooperates with chalcone isomerase and chalcone synthase to form different anthocyanins, an important step in flower color formation (Winkel-Shirley and Flavonoid, 2001). PAT proteins are members of lipid droplet proteins family that regulates cellular lipid storage. It may play an important role in the rapid synthesis of lipid droplets, the stabilization of nascent lipid droplets, the regulation of lipid droplet maturation, and the intracellular transport of lipid droplets (Wolins et al., 2005). 4-coumarate: CoA ligase (4CL) regulates plant lignin metabolism and is involved in the synthesis of flavonoids and other secondary metabolites synthesis. Studies have shown that 4CL is important for cell wall formation, water transport, plant stress resistance, and disease resistance (Chen et al., 2019b; Geng et al., 2020; Xiao et al., 2021). There are also some missing annotated proteins in the interaction network map. They have obvious direct or indirect synergistic effects with SlPALs and SpenPALs proteins, but their functions remain unclear.

### 3.10 Gene co-expression module construction

**Figure 9:**
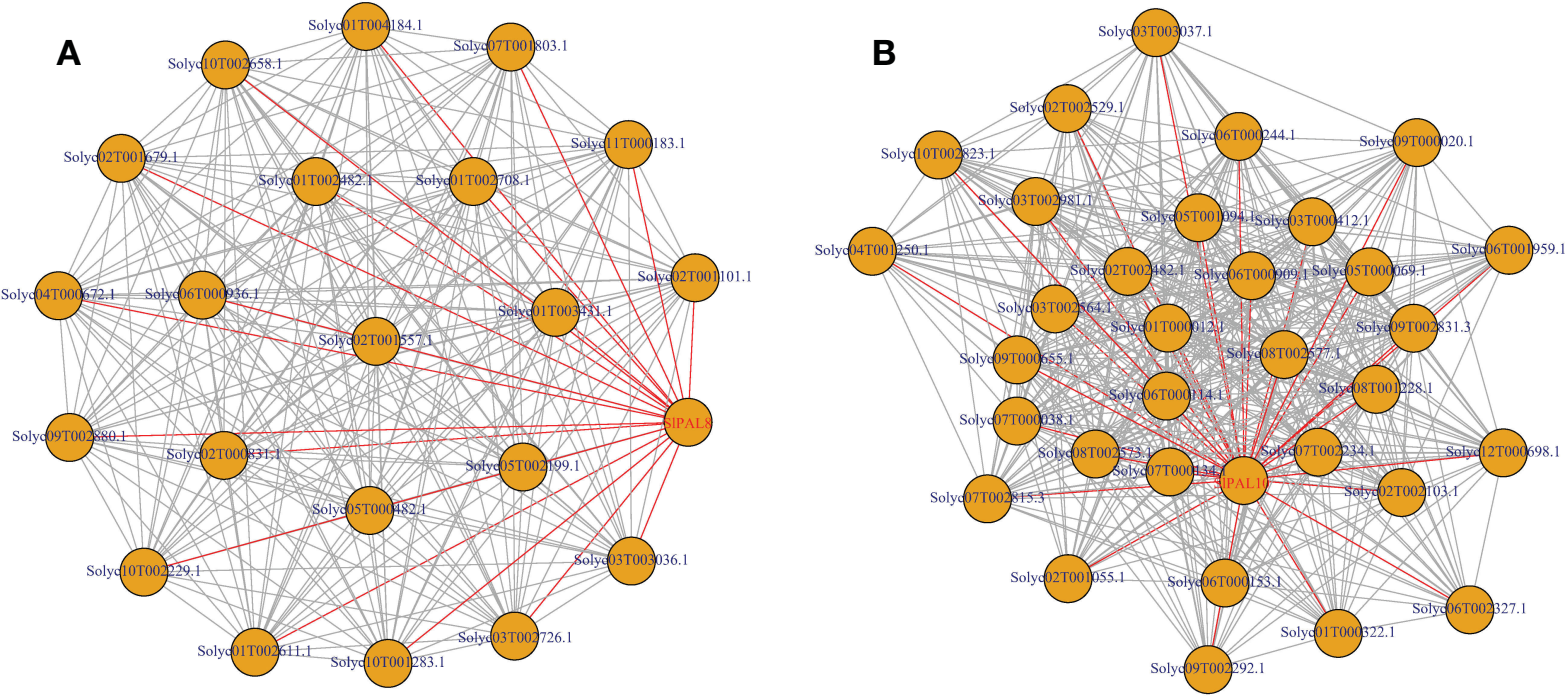
SlPAL8 and SlPAL10 module key gene interaction network **(A)**: *SlPAL8*; **(B)**: *SlPAL10*. Genes with edge weights of >0.1 were visualized using Cytoscape, with each node representing a gene and connecting lines between genes indicating co-expression correlations. Co-lines between the *SlPAL* gene and other genes are indicated by red lines.

To identify genes associated with SlPAL expression patterns, we performed a weighted gene co-expression network analysis (WGCNA) using tomato resistance and fruit development-related RNA-seq sequencing data (PRJNA888477). A total of 18224 tomato genes with expression levels (FPKM) ≥10 in at least one sample were calculated for the construction of WGCNA (**Figure 9**). The results showed that there are two PAL genes present in separate co-expression modules, module A contains 21 co-expressed genes, of which Solyc01T002708.1 and Solyc11T000183.1 have mangiferin biosynthesis function, implying that PAL is involved in mangiferin synthesis together with these genes. Module B contains 32 co-expressed genes, of which Solyc06T000114.1 and Solyc09T002292.1 have successful functions in encoding enzymes and proteins, implying that PAL is involved in enzyme and protein synthesis together with these genes. The genes with the highest connectivity in the 2 modules were selected as the core genes and mapped separately for the interactions network. the functional annotation of the 53 core genes in the 2 key modules is shown in (**Supplementary Table S2**).

### 3.11 Expression of tomato *PAL* genes after root-knot nematode infection

**Figure 10:**
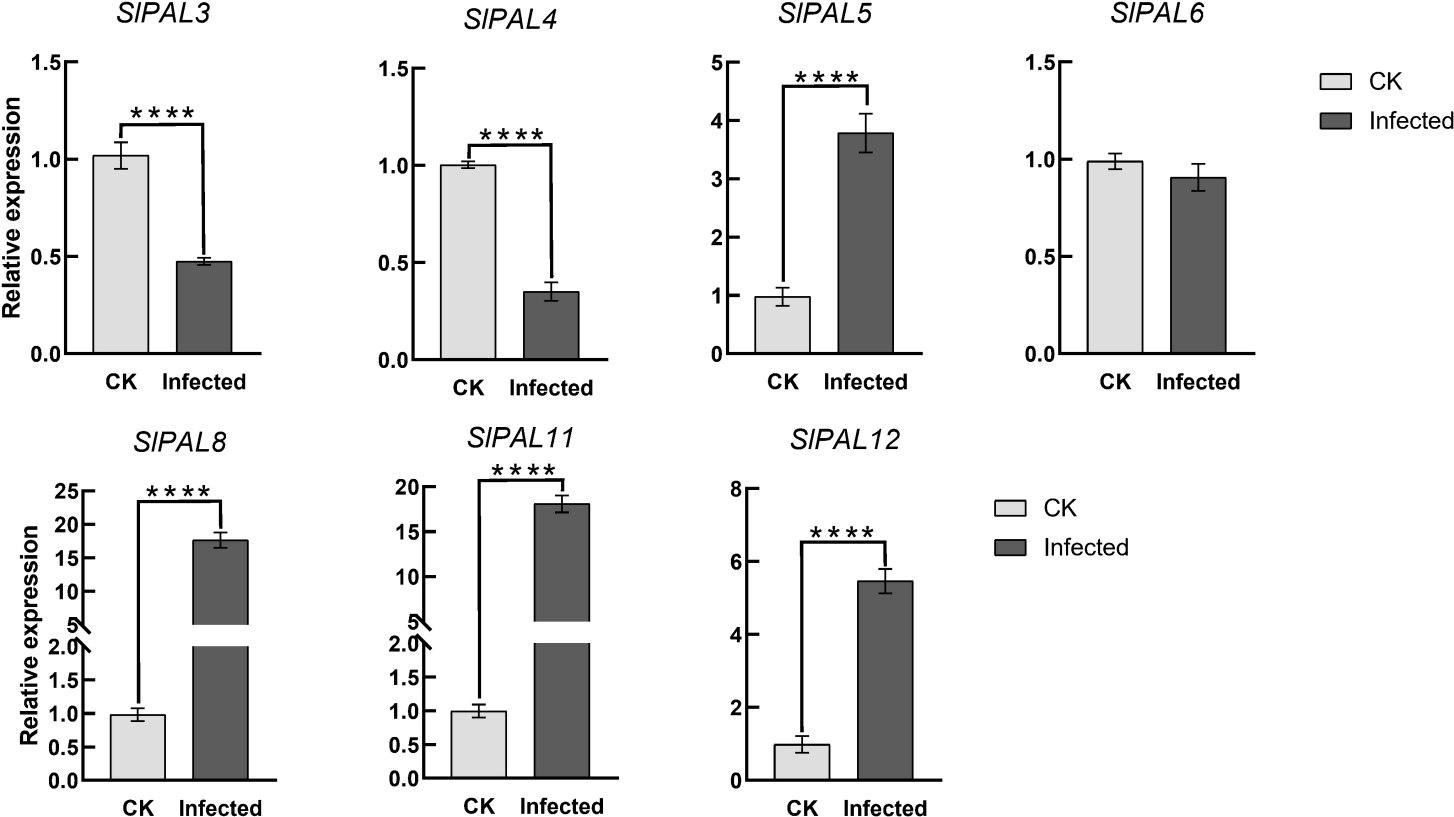
qRT-PCR analysis of *SlPAL* genes under root-knot nematode treatment CK: control group, naturally grown tomatoes without any treatment. Infected: tomatoes infected with root knot nematodes for 5 days. Data represent the means ± SD of four biological replicates, The asterisk represents a significant difference compared with the control based on Student*’*s *t*-test (*p < 0.05), ****: p-value<0.0001.

To verify the potential role of SlPALs under root-knot nematode infection, their expression levels were analyzed by qRT-PCR (**Figure 10**). It could be found that after root-knot nematode infection, the expression of SlPAL5, SlPAL8, SlPAL11, and SlPAL12 genes were higher than the control, and the expression of SlPAL3, SlPAL4, and SlPAL6 genes were lower than the control.

## 4 Discussion

Secondary metabolism is the result of long-term evolutionary adaptation of plants to the ecological environment, and it plays an important role in dealing with the relationship between plants and the ecological environment. It enables plants to better adapt to their environment during development and to change themselves to form their unique individual characteristics (Dangl and Jones, 2001). As a key secondary metabolic pathway, phenylpropane metabolism begins with phenylalanine and eventually leads to the production of secondary metabolites such as flavonoids, phenols and lignans (Wang et al., 2016; Zhan et al., 2022). PAL, as an enzyme in the first reaction step of phenylpropane metabolism, plays a key and has a major impact on the subsequent secondary metabolism (Wei et al., 2023). The number of PAL family members currently varies among plants, e.g., four PAL genes were identified in Arabidopsis, 13 in maize, nine in rice, and four in citrus. 14 and 11 were identified in cultivated tomato and S pennellii, respectively, in the present study, with numbers similar to those of the monocotyledonous plant maize. The number of *SlPAL* and *SpenPAL* genes far exceeded that of Arabidopsis, suggesting that tomato may have undergone genomic duplication events occurred during evolution (Wanner et al., 1995; Raes et al., 2003), and tandem and fragmental duplication can be seen to have occurred in cultivated tomato and *S. pennellii* based on chromosomal localization and intraspecific co-linearity (**Figures 1**, **7**). Replication events are important in the expansion and evolution of gene families and include whole genome replication, small fragment replication, local tandem replication or a combination of these possibilities (Ober, 2005; Lei et al., 2012; Xue et al., 2012).

Physicochemically, both SlPALs and SpenPALs are basically acidic hydrophobic proteins, which are also similar to the reported PAL genes of other plants (Zhan et al., 2022; Wei et al., 2023). Most of the SpenPALs have around 700 amino acids, while the number of amino acids in SlPALs varies widely. According to previous reports, there is a clear separation between monocotyledonous and dicotyledonous PAL genes in the phylogenetic tree (Sun et al., 2016), and this distribution also appeared in this study, and their functional differentiation may have been formed before the differentiation of monocotyledonous and dicotyledonous plants. The functions of genes and their structures are inextricably linked, and most of the SlPAL and SpenPAL genes contain a highly conserved methylene imidazolinone (MIO) electrophilic motif, a characteristic sequence of the PAL gene family (Song and Wang, 2009; Chen, 2019).

CHS is associated with the synthesis of flavonoids and phenolics and is the first rate-limiting enzyme in the plant flavonoid synthesis pathway. CHS and F3H are important steps in flower color formation and both are closely related to anthocyanin synthesis and metabolism (Dong and Lin, 2021). In Arabidopsis, AtMYB75 leads to increased expression of related PAL genes, resulting in elevated anthocyanin content (Bhargava et al., 2010). AtPAL1 and AtPAL2 are highly expressed in roots and mature flowers, but hardly expressed in leaf tissue (Wanner et al., 1995; Raes et al., 2003), thus suggesting that PAL genes have an important role in the control of flower color. It is evident through protein interactions that PAL genes are indeed inextricably linked to the production of various secondary metabolites such as lignin and flavonoids. When plants are subjected to external biotic persecution, the process of phenylpropanoid synthesis is activated, resulting in a rapid increase in PAL enzyme activity, which improves plant tolerance to biotic stress by producing secondary metabolites such as lignin (Dixon and Paiva, 1995; Chezem et al., 2017; Zhou et al., 2018). 4CL regulates plant lignin metabolism, lignin enhances cell walls, enhances tissue lignification, and forms a mechanical barrier against invasion by pathogenic bacteria (Liu et al., 2018), and in rice, PAL1 affects resistance to rice blast fungus by altering lignin content (Solekha et al., 2020).

The ability of tomato to respond to stress directly affects its yield and quality, thus it is important to explore stress-responsive genes in wild sources. Previous studies have shown that PAL genes from different plants are widely involved in the regulation of various biotic stresses (Zhan et al., 2022; Wei et al., 2023). These studies have mainly focused on the relationship between the products of this metabolic pathway and plant root-knot nematode resistance. Phytochemicals have toxic and tropic effects on herbivorous root-knot nematode, and there is an indirect relationship between the production of these compounds and PAL content. Numerous experiments have demonstrated that the level of PAL activity is strongly related to plant root-knot nematode resistance and that feeding by herbivores can also induce high PAL activity (Gao, 2009). However, there are fewer reports on the direct involvement of PAL in root-knot nematode resistance in tomato. We treated tomatoes with root-knot nematodes, resulting in qRT-PCR analysis of the SlPAL genes family (**Figure 10**). The results showed that SlPAL5, SlPAL8, SlPAL11, and SlPAL12 genes responded positively to root-knot nematode infection and that the expression of SlPAL8 and SlPAL11 was approximately 18-fold higher than that of the control. Interestingly SlPAL3 and SlPAL4 underwent a significant negative regulation, probably because these two genes are not involved in the infection of root-knot nematodes. Our qRT-PCR results further conclude that the SlPAL partial gene family is positively regulated for root-knot nematode infection.

## 5 Conclusion

In this study, the composition of tomato PAL genes was identified at the genome-wide level by bioinformatics methods, and a total of 11 and 14 PAL genes were identified from S. pennellii and cultivated tomatoes and analyzed for their physicochemical properties, phylogenetic relationships, gene structure, covariance, promoter elements, and protein interactions. Expression analysis of tomato under root-knot nematode resistance treatment based on qRT-PCR assay revealed that SlPAL genes were significantly expressed in response to root-knot nematode treatment. Thus, it provides a theoretical basis for an in-depth study of tomato PAL family members under biotic stresses.

## Data availability statement

Publicly available datasets were analyzed in this study. This data can be found here: PRJNA888477.

## Author contributions

NL and QY guided the design of the experiment. FZ and JW directed the data analysis. FZ and XL conducted data analysis, FZ and JW manuscript writing. JZ, YL and YC finished plant material handling, NL and QY supervised the experiment and confirmed the manuscript. All authors contributed to the article and approved the submitted version. Thank all the above staff for the help in this study.

## References

[B1] Artimo P. Jonnalagedda M. Arnold K. Baratin D. Csardi G. de Castro E.. (2012). ExPASy: SIB bioinformatics resource portal. Nucleic Acids Res. 40, W597–W603. doi: 10.1093/nar/gks400 22661580 PMC3394269

[B2] Bhargava A. Mansfield S. D. Hall H. C. Douglas C. J. Ellis B. E. (2010). *MYB75* functions in regulation of secondary cell wall formation in the arabidopsis inflorescence stem. Plant Physiol. 154, 1428–1438. doi: 10.1104/pp.110.162735 20807862 PMC2971618

[B3] Chaman M. E. Copaja S. V. Argandoña V. H. (2003). Relationships between salicylic acid content, phenylalanine ammonia-lyase (PAL) activity, and resistance of barley to aphid infestation. J. Agric. Food Chem. 51, 2227–2231. doi: 10.1021/jf020953b 12670161

[B4] Chen X. (2019). Cloning and expression analysis of phenylalanine ammonia-lyase gene in lonicera macranthoides. Chin. Tradit. Herb. Drugs 24, 178–187. doi: 10.7501/j.issn.0253-2670.2019.01.027

[B5] Chen C. Chen H. Zhang Y. Thomas H. R. Frank M. H. He Y.. (2020). TBtools: an integrative toolkit developed for interactive analyses of big biological data. Mol. Plant 13, 1194–1202. doi: 10.1016/j.molp.2020.06.009 32585190

[B6] Chen X. Wang H. Li X. Ma K. Zhan Y. Zeng F. (2019b). Molecular cloning and functional analysis of 4-coumarate: CoA ligase 4 (4CL-like 1) from fraxinus mandshurica and its role in abiotic stress tolerance and cell wall synthesis. BMC Plant Biol. 19, 231. doi: 10.1186/s12870-019-1812-0 31159735 PMC6545724

[B7] Chezem W. R. Memon A. Li F. S. Weng J. K. Clay N. K. (2017). SG2-type R2R3-MYB transcription factor *MYB15* controls defense-induced lignification and basal immunity in arabidopsis. Plant Cell. 29, 1907–1926. doi: 10.1105/tpc.16.00954 28733420 PMC5590497

[B8] Costa M. A. Collins R. E. Anterola A. M. Cochrane F. C. Davin L. B. Lewis N. G. (2003). An insilico assessment of gene function and organization of the phenylpropanoid pathway metabolic networks in *Arabidopsis thaliana* and limitations thereof. Phytochemistry 64, 1097–1112. doi: 10.1016/s0031-9422(03)00517-x 14568076

[B9] Dangl J. L. Jones J. D. (2001). Plant pathogens and integrated defence responses to infection. Nature 411, 826–833. doi: 10.1038/35081161 11459065

[B10] Dao T. T. Linthorst H. J. Verpoorte R. (2011). Chalcone synthase and its functions in plant resistance. Phytochem. Rev. 10, 397–412. doi: 10.1007/s11101-011-9211-7 21909286 PMC3148432

[B11] Deng L. C. Cui L. N. Yang L. Chen J. He W. Z. Li X.. (2019). Identification of gene family of phenylalanine ammonia-lyase and analysis of resistance to maize sheath blight in corn. Mol. Plant Breed. 03, 891–897. doi: 10.13271/j.mpb.017.000891

[B12] Dixon R. A. Paiva N. L. (1995). Stress-induced phenylpropanoid metabolism. Plant Cell. 7, 1085–1097. doi: 10.1105/tpc.7.7.1085.1 12242399 PMC160915

[B13] Dong N. Q. Lin H. X. (2021). Contribution of phenylpropanoid metabolism to plant development and plant-environment interactions. J. Integr. Plant Biol. 63, 180–209. doi: 10.1111/jipb.13054 33325112

[B14] Dong C. J. Shang Q. M. (2013). Geno me-wide characterization of phenylalanine ammonia-lyase gene family in watermelon (*Citrullus lanatus*). Planta 238, 35–49. doi: 10.1007/s00425-013-1869-1 23546528

[B15] Eddy S. R. (2008). A probabilistic model of local sequence alignment that simplifies statistical significance estimation. PloS Comput. Biol. 4, e1000069. doi: 10.1371/journal.pcbi.1000069 18516236 PMC2396288

[B16] Fernandez-Pozo N. Menda N. Edwards J. D. Saha S. Tecle I. Y. Strickler S. R.. (2015). The sol genomics network (SGN)-from genotype to phenotype to breeding. Nucleic Acids Res. 43, D1036–D1041. doi: 10.1093/nar/gku1195 25428362 PMC4383978

[B17] Finn R. D. Clements J. Eddy S. R. (2011). HMMER web server: interactive sequence similarity searching. Nucleic Acids Res. 39, W29–W37. doi: 10.1093/nar/gkr367 21593126 PMC3125773

[B18] Fraser C. M. Chapple C. (2011). The phenylpropanoid pathway in arabidopsis. Arabidopsis Book 9, e0152. doi: 10.1199/tab.0152 22303276 PMC3268504

[B19] Gao X. (2009). Research progress in plant phenylalanine ammonia lyases. Modern Agric. Sci. Technol 4, 1007–5739. doi: 10.3969/j.issn

[B20] Geng P. Zhang S. Liu J. Zhao C. Wu J. Cao Y.. (2020). *MYB20*, *MYB42*, *MYB43*, and *MYB85* regulate phenylalanine and lignin biosynthesis during secondary cell wall formation. Plant Physiol. 182, 1272–1283. doi: 10.1104/pp.19.01070 31871072 PMC7054866

[B21] Goodstein D. M. Shu S. Howson R. Neupane R. Hayes R. D. Fazo J.. (2012). Phytozome: a comparative platform for green plant genomics. Nucleic Acids Res. 40, D1178–D1186. doi: 10.1093/nar/gkr944 22110026 PMC3245001

[B22] Grundy W. N. Bailey T. L. Elkan C. P. Baker M. E. (1997). Meta-MEME: motif-based hidden markov models of protein families. Bioinformatics 13, 397–406. doi: 10.1093/bioinformatics/13.4.397 9283754

[B23] Horton P. Park K. J. Obayashi T. Fujita N. Harada H. Adams-Collier C. J.. (2007). WoLF PSORT: protein localization predictor. Nucleic Acids Res. 35, W585–W587. doi: 10.1093/nar/gkm259 17517783 PMC1933216

[B24] Huang J. Gu M. Lai Z. Fan B. Shi K. Zhou Y. H.. (2010). Functional analysis of the arabidopsis *PAL* gene family in plant growth, development, and response to environmental stress. Plant Physiol. 153, 1526–1538. doi: 10.1104/pp.110.157370 20566705 PMC2923909

[B25] Jiang W. Q. Yang L. He Y. Q. Zhang H. T. Li W. Chen H. G.. (2019). Genome-wide identification and transcriptional expression analysis of superoxide dismutase (SOD) family in wheat (*Triticum aestivum*). Peer J. 7, e8062. doi: 10.7717/peerj.8062 31763072 PMC6873880

[B26] Kumar S. Stecher G. Tamura K. (2016). MEGA7: molecular evolutionary genetics analysis version 7.0 for bigger datasets. Mol. Biol. Evol. 33, 1870–1874. doi: 10.1093/molbev/msw054 27004904 PMC8210823

[B27] Lei L. Zhou S. L. Ma H. Zhang L. S. (2012). Expansion and diversification of the set domain gene family following whole-genome duplications in *Populus trichocarpa* . BMC Evol. Biol. 12, 51. doi: 10.1186/1471-2148-12-51 22497662 PMC3402991

[B28] Li W. Yang Y. Qiao C. Zhang G. Luo Y. (2018). Functional characterization of phenylalanine ammonia-lyase and cinnamate 4-hydroxylase-encoding genes from *Lycoris radiata*, a galanthamine-producing plant. Int. J. Biol. Macromol. 117, 1264–1279. doi: 10.1016/j.ijbiomac 29894786

[B29] Liu Q. Luo L. Zheng L. (2018). Lignins: biosynthesis and biological functions in plants. Int. J. Mol. Sci. 19, 335. doi: 10.3390/ijms19020335 29364145 PMC5855557

[B30] Liu X. L. Yin Y. H. Gao Y. D. Li K. (2022). *SlNPF68* gene involved in root foraging response under nitrogen deficiency in tomato. J. Plant Physiol. 58, 1212–1220. doi: 10.13592/j.cnki.ppj.100178

[B31] Livak K. J. Schmittgen T. D. (2001). Analysis of relative gene expression data using real-time quantitative PCR and the 2^–ΔΔCT^ method. Methods 25, 402–408. doi: 10.1006/meth.2001.1262 11846609

[B32] MacDonald M. J. D’Cunha G. B. (2007). A modern view of phenylalanine ammonia lyase. Biochem. Cell Biol. 85, 273–282. doi: 10.1139/O07-018 17612622

[B33] Nugroho L. H. Verberne M. C. Verpoorte R. (2002). Activities of enzymes involved in the phenylpropanoid pathway in constitutively salicylic acid-producing tobacco plants. Plant Physiol. Biochem. 40, 755–760. doi: 10.1016/S0981-9428(02)01437-7

[B34] Ober D. (2005). Seeing double: gene duplication and diversification in plant secondary metabolism. T rends Plant Sci. 10, 444–449. doi: 10.1016/j.tplants.2005.07.007 16054418

[B35] Olsen K. M. Lea U. S. Slimestad R. Verheul M. Lillo C. (2008). Differential expression of four arabidopsis *PAL* genes; *PAL1* and *PAL2* have functional specialization in abiotic environmental triggered flavonoid synthesis. J. Plant Physiol. 165, 1491–1499. doi: 10.1016/j.jplph.2007.11.005 18242769

[B36] Raes J. Rohde A. Christensen J. H. Van Y. D. P. Boerjan W. (2003). Genome-wide characterization of the lignification toolbox in arabidopsis. Plant Physiol. 133, 1051–1071. doi: 10.1104/pp.103.026484 14612585 PMC523881

[B37] Ritter H. Schulz G. E. (2004). Structural basis for the entrance into the phenylpropanoid metabolism catalyzed by phenylalanine ammonia-lyase. Plant Cell. 16, 3426–3436. doi: 10.1105/tpc.104.025288 15548745 PMC535883

[B38] Rombauts S. Déhais P. Van Montagu M. Rouzé P. (1999). PlantCARE, a plant cis acting regulatory element database. Nucleic Acids Res. 27, 295–296. doi: 10.1093/nar/27.1.295 9847207 PMC148162

[B39] Schultz J. Copley R. R. Doerks T. Ponting C. P. Bork P. (2000). SMART: a web-based tool for the study of genetically mobile domains. Nucleic Acids Res. 28, 231–234. doi: 10.1093/nar/28.1.231 10592234 PMC102444

[B40] Shi R. Shuford C. M. Wang J. P. Sun Y. H. Yang Z. Chen H. C.. (2013). Regulation of phenylalanine ammonia-lyase (*PAL*) gene family in wood forming tissue of populus. trichocarpa. Planta. 238, 487–497. doi: 10.1007/s00425-013-1905-1 23765265

[B41] Solekha R. Susanto F. A. Joko T. Nuringtyas T. R. Purwestri Y. A. (2020). Phenylalanine ammonia lyase (PAL) contributes to the resistance of black rice against xanthomonas oryzae pv. oryzae. J. Plant Pathol. 102, 359–365. doi: 10.1007/s42161-019-00426-z

[B42] Song D. D. Shi M. X. Gao J. H. Mi Q. H. Duan L. Zhuang X.. (2022). Optical properties of complex-type of light conversion greenhouse films and their effects on the growth and fruit qualities of tomato in solar greenhouse. J. Plant Physiol. 58, 2218–2226. doi: 10.13592/j.cnki.ppj.100145

[B43] Song J. Wang Z. (2009). Molecular cloning, expression and characterization of a phenylalanine ammonialyase gene (*SmPAL1*) from *Salvia miltiorrhiza* . Mol. Biol. Rep. 36, 939–952. doi: 10.1007/s11033-008-9266-8 18454352

[B44] Sonnhammer E. L. L. Eddy S. R. Durbin R. (1997). Pfam: a comprehensive database of protein domain families based on seed alignments. Proteins 28, 405–420. doi: 10.1002/(sici)1097-0134(199707)28:3<405:aid-prot10>3.0.co;2-l 9223186

[B45] Sun R. Z. Zhang X. Chen G. Li Q. Zhu Y. R. Chen W.. (2016). Genome wide identification and expression analysis of grape phenylalanine ammonia lyase gene family. J. Plant Physiol. 52, 195–208. doi: 10.13592/j.cnki.ppj

[B46] Szklarczyk D. Gable A. L. Lyon D. Junge A. Wyder S. Huerta-Cepas J.. (2019). STRING V11: protein-protein association networks with increased coverage, supporting functional discovery in genome-wide experimental datasets. Nucleic Acids Res. 47, D607–D613. doi: 10.1093/nar/gky1131 30476243 PMC6323986

[B47] Wang Z. Li J. Y. Jia C. H. Li J. P. Xu B. Y. Jin Z. Q. (2016). Molecular cloning and expression of four phenylalanine ammonia lyase genes from banana interacting with fusarium oxysporum. Biol. Plant 60, 459–468. doi: 10.1007/s10535-016-0619-1

[B48] Wang D. Zhang Y. Zhang Z. Zhu J. Yu J. (2010). KaKs_calculator 2.0: a toolkit incorporating gamma-series methods and sliding window strategies. Genom. Proteomics Bioinf. 8, 77–80. doi: 10.1016/S1672-0229(10)60008-3 PMC5054116 20451164

[B49] Wanner L. A. Li G. Ware D. Somssich I. E. Davis K. R. (1995). The phenylalanine ammonia-lyase gene family in *Arabidopsis thaliana* . Plant Mol. Biol. 27, 327–338. doi: 10.1007/BF00020187 7888622

[B50] Wei L. L. Wang W. J. Li T. Chen O. Yao S. X. Deng L. L.. (2023). Genome-wide identification of the *CsPAL* gene family and functional analysis for strengthening green mold resistance in citrus fruit. Postharvest Biol. Tec. 196, 112178–91. doi: 10.1016/J.POSTHARVBIO.2022.112178

[B51] Winkel-Shirley B. Flavonoid B. (2001). A colorful model for genetics, biochemistry, cell biology, and biotechnology. Plant Physiol. 126, 485–493. doi: 10.1104/pp.126.2.485 11402179 PMC1540115

[B52] Wolins N. E. Quaynor B. K. Skinner J. R. Schoenfish M. J. Tzekov A. Bickel P. E. (2005). S3-12, adipophilin, and TIP47 package lipid in adipocytes. J. Biol. Chem. 280, 19146–19455. doi: 10.1074/jbc.M500978200 15731108

[B53] Xiao S. Hu Q. Shen J. Liu S. Yang Z. Chen K.. (2021). *GhMYB4* downregulates lignin biosynthesis and enhances cotton resistance to verticillium dahliae. Plant Cell Rep. 40, 735–751. doi: 10.1007/s00299-021-02672-x 33638657

[B54] Xu X. X. Yang S. G. (2009). Advances in the studies of phenylalanine ammonialyase. J. Anhui AG Sci. 37, 15115–15119. doi: 10.13989/j.cnki.0517-6611

[B55] Xue Z. Duan L. Liu D. Guo J. Ge S. Dicks J.. (2012). Divergent evolution of oxidosqualene cyclases in plants. New Phytol. 193, 1022–1038. doi: 10.1111/j.1469-8137 22150097

[B56] Yan F. Li H. Zhao P. (2019). Genome-wide identification and transcriptional expression of the *PAL* gene family in common walnut (*Juglansregia l.*). Genes 10, 46. doi: 10.3390/genes10010046 30650597 PMC6357058

[B57] Zeng J. L. Ouyang L. J. Liu J. L. He H. H. Zhu C. L. Peng X. S.. (2018). Whole genome analysis and stress expression research of pal gene in rice. Genomics Appl. Biol. 9, 3881–3888. doi: 10.13417/j.gab.037.003881

[B58] Zhan C. Li Y. Li H. Wang M. Gong S. Ma D.. (2022). Phylogenomic analysis of phenylalanine ammonia-lyase (PAL) multigene family and their differential expression analysis in wheat (*Triticum aestivum* l.) suggested their roles during different stress responses. Front. Plant Sci. 30. doi: 10.3389/fpls.2022.982457 PMC9561908 36247561

[B59] Zhang H. Huang Q. Yi L. Song X. Li L. Deng G. (2021). Pal-mediated SA biosynthesis pathway contributes to nematode resistance in wheat. Plant J. 107, 698–712. doi: 10.1111/tpj.15316 33974322

[B60] Zhou Y. Ma J. Xie J. Deng L. Yao S. Zeng K. (2018). Transcriptomic and biochemical analysis of highlighted induction of phenylpropanoid pathway metabolism of citrus fruit in response to salicylic acid, pichia membranaefaciens and oligochitosan. Postharvest Biol. Technol. 142, 81–92. doi: 10.1016/j.postharvbio

