# Supplementary material for: Genome-wide identification and expression analyses of phenylalanine ammonia-lyase gene family members from tomato (Solanum lycopersicum) reveal their role in root-knot nematode infection
Source: Front Plant Sci. 2023 Jun 6;14:1204990. doi: 10.3389/fpls.2023.1204990 (PMC10280380; doi:10.3389/fpls.2023.1204990)
Supplement: Supplementary file 1 [file DataSheet_1.pdf]

Supplementary Material

Genome-wide identification and expression analysis under root-knot nematode treatment of the Phenylalanine ammonia-lyase gene family in tomato (*Solanum lycopersicum*)

Fuling Zhang[1,2], Tao Yang[1], Xianguo Li[1,2], Jun Zhang[3], Yuxiang Liu[1,2], Yijia Chen[1,2], Qinghui Yu[1*], Ning Li[1*]

***Corresponding author**

Ning Li

lining@xaas.ac.cn

Qinghui Yu

yuqinghui@xaac.ac.cn

**Supplementary TABLE 1 | ** Primers sequences used for qRT-PCR.

Gene name	Forward primer sequence (5′ →3′)	Reverse primer sequence (5′ →3′)
SlBURP3	TGAATGGCACAGCAGTTGG	AGCCTCAATCTGACCAGGGT
SlBURP4	TGAATGGCACAGCAGTTGG	AGCAGCCTCAATCTGACCAG
SlBURP5	TGCTTCAAGGTTACTCTGGCA	TCAAGTTTCTCACCATTGGGTC
SlBURP6	TGCTCACTGGCAAGCCTAAT	GGGTTTCCCGTTCATCACTT
SlBURP8	AGGTGCTGAAATCGCTATGG	TGTCAACTGCCTCCTCTGTCT
SlBURP11	TGCCATCTAATCTCACAGGAGG	AAGCCCAAGGAGTTCACATCT
SlBURP12	GGTTGCCATCTAATCTCACAGG	AAGCCCAAGGAGTTCACATCT
SlActin	CGGTGACCACTTTCCGATCT	TCCTCACCGTCAGCCATTTT

Supplementary TABLE 2 | Functional annotation of core genes in key modules

Module	Hub gene	Gene description
A	*Solyc01T004184.1*	BEL1-like homeodomain 7
	Solyc10T002658.1	First enzyme in the biosynthetic pathway of isoleucine
	Solyc07T001803.1	Putative cytochrome P450
	Solyc02T001679.1	Quinone reductase family protein
	Solyc01T002482.1	Belongs to a clade of five Solanum lycopersicum ABCG half-Transporters that are required for synthesis of an effective suberin barrier in roots and seed coats
	Solyc01T002708.1	5-enolpyruvylshikimate-3-phosphate synthase involved in shikimic acid biosynthesis
	Solyc11T000183.1	5-enolpyruvylshikimate-3-phosphate synthase involved in shikimic acid biosynthesis
	Solyc04T000672.1	ATP-binding cassette A1
	Solyc06T000936.1	A senescence-associated gene whose expression is induced in Response to treatment with Nep1, a fungal protein that causes necrosis
	Solyc02T001557.1	Ubiquitin-conjugating enzyme family protein
	Solyc01T003431.1	Encodes a S-adenosylmethionine synthetase
	Solyc02T001101.1	ATP dependent copper transporter vital for ethylene response pathway
	Solyc09T002880.1	Unknown Protein
	Solyc02T000831.1	Hydroxyproline-rich glycoprotein family protein
	Solyc05T000482.1	Transmembrane amino acid transporter family protein
	Solyc05T002199.1	Transketolase involved in carbon fixation
	Solyc10T002229.1	Encodes an auxin efflux carrier involved in shoot and root development
	Solyc01T002611.1	Encodes VAD1 (Vascular Associated Death1)
	Solyc10T001283.1	Involved in multiple stress signaling pathways; negatively regulates RPW8.1-mediated cell death and disease resistance

	Solyc03T002726.1	Encodes a serine/threonine protein kinase
	Solyc03T003036.1	Encode for the hydroxycinnamoyl-Coenzyme A shikimate/quinate hydroxycinnamoyltransferase (HCT)
B	*Solyc03T003037.1*	Heat shock protein 70B
	Solyc02T002529.1	Molybdenum cofactor synthesis family protein
	Solyc10T002823.1	Encodes the tomato homolog of a conserved eukaryotic protein without known functional domains
	Solyc06T000244.1	TolB protein-like protein
	Solyc09T000020.1	Ubiquitin carboxyl-terminal hydrolase
	Solyc03T002981.1	Transmembrane protein
	Solyc04T001250.1	Unknown Protein
	Solyc05T001094.1	Unknown Protein
	Solyc03T000412.1	AtRBL2 has been identified as a rhomboid protein involved in regulated intramembrane proteolysis (RIP)
	Solyc06T001959.1	Duplicated homeodomain-like superfamily protein
	Solyc02T002482.1	Alpha/beta-Hydrolases superfamily protein
	Solyc06T000909.1	Encodes a member of the PYR (pyrabactin resistance)
	Solyc05T000069.1	Encodes an enzyme putatively involved in trehalose biosynthesis
	Solyc03T002564.1	Galactose oxidase/kelch repeat superfamily protein
	Solyc01T000012.1	Encodes a subunit of the dolichol phosphate mannase synthase (DPMS)
	Solyc09T002831.3	NAD(P)-linked oxidoreductase superfamily protein
	Solyc09T000655.1	HXXXD-type acyl-transferase family protein
	Solyc08T002577.1	GroES-like zinc-binding dehydrogenase family protein
	Solyc06T000114.1	Encodes a glutamine-dependent asparagine synthetase
	Solyc07T000038.1	Encodes a basic helix-loop-helix (bHLH) protein that regulates root hair and sperm cell development

Solyc08T002573.1	Member of IQ67 (CaM binding) domain containing family
Solyc07T000134.1	Unknown Protein
Solyc07T002234.1	Member of WRKY Transcription Factor
Solyc12T000698.1	O-Glycosyl hydrolases family 17 protein
Solyc02T002103.1	Contains Interpro domain
Solyc07T002815.3	NAI1 interacting protein
Solyc02T001055.1	ABA-induced transcription repressor that acts as feedback regulator in ABA signalling
Solyc06T000153.1	Actoylglutathione lyase / glyoxalase I family protein
Solyc09T002292.1	Encodes a DNA binding protein with transcription activation activity. It is expressed in response to osmotic, drought and ABA stress
Solyc01T000322.1	Encodes a nuclear protein that binds to RNA with a specificity for oligouridylates in vitro
Solyc06T002327.1	SRPK-family member protein
Solyc08T001228.1	CGEP | chloroplast glutamyl peptidase
